# DNA Methylome and LncRNAome Analysis Provide Insights Into Mechanisms of Genome-Dosage Effects in Autotetraploid Cassava

**DOI:** 10.3389/fpls.2022.915056

**Published:** 2022-07-04

**Authors:** Liang Xiao, Liuying Lu, Wendan Zeng, Xiaohong Shang, Sheng Cao, Huabing Yan

**Affiliations:** ^1^Cash Crops Research Institute, Guangxi Academy of Agricultural Sciences, Nanning, China; ^2^State Key Laboratory for Conservation and Utilization of Subtropical Agro-Bioresources, Guangxi University, Nanning, China

**Keywords:** autotetraploid cassava, genome-dosage effect, DNA-methylation, lncRNA expression, protein coding gene expression, transposon

## Abstract

Whole genome duplication (WGD) increases the dosage of all coding and non-coding genes, yet the molecular implications of genome-dosage effects remain elusive. In this study, we generated integrated maps of the methylomes and lncRNAomes for diploid and artificially generated autotetraploid cassava (*Manihot esculenta* Crantz). We found that transposable elements (TEs) suppressed adjacent protein coding gene (PCG)-expression levels, while TEs activated the expression of nearby long non-coding RNAs (lncRNAs) in the cassava genome. The hypermethylation of DNA transposons in mCG and mCHH sites may be an effective way to suppress the expression of nearby PCGs in autotetraploid cassava, resulting in similar expression levels for most of PCGs between autotetraploid and diploid cassava. In the autotetraploid, decreased methylation levels of retrotransposons at mCHG and mCHH sites contributed to reduced methylation of Gypsy-neighboring long intergenic non-coding RNAs, potentially preserving diploid-like expression patterns in the major of lncRNAs. Collectively, our study highlighted that WGD-induced DNA methylation variation in DNA transposons and retrotransposons may be as direct adaptive responses to dosage of all coding-genes and lncRNAs, respectively.

## Introduction

Polyploidy or whole genome duplication (WGD), is the heritable condition of possessing multiple sets of chromosomes co-occurring in a nucleus, with four being the most common (Comai, 2005; Madlung and Wendel, 2013). During the last several decades, there has been a resurgence of interest in the study of polyploid evolution, particularly the genetic and genomic consequences of polyploidy (Gaeta et al., 2007; Salmon et al., 2010; Shi et al., 2012; Renny-Byfield and Wendel, 2014; Visger et al., 2019). Polyploidy can result in considerable changes in both coding and non-coding gene expression caused by the increasing of the number of alleles at each locus, which provides a molecular basis for adaptation (Doyle et al., 2008; Salmon and Ainouche, 2010; Zhang et al., 2019; Tao et al., 2021). Two forms of polyploidy are often considered in plants (Ramsey and Schemske, 1998): allopolyploidy species are traditionally considered to arise *via* interspecific hybridization and subsequent doubling of non-homologous genomes (AABB) (Xu et al., 2009; Kraitshtein et al., 2010), whereas autopolyploids arise within a single species by doubling of structurally similar, homologous genomes (AAAA) (Havananda et al., 2011; Liu and Sun, 2017). Although past views pointed that autopolyploidy is likely rare, increasing evidences indicated that autopolyploid taxa might be more common and the appearance of autotetraploidy plants in nature might be significantly underestimated (Ramsey and Schemske, 2002; Soltis et al., 2003; Soltis and Soltis, 2009; Parisod et al., 2010; Barker et al., 2016), despite potential weaknesses, such as sterility, aneuploidy, genomic and epigenetic instabilities (Comai, 2005; Madlung and Wendel, 2013). In newly formed polyploids, genome doubling events increase the dosage of all coding and non-coding genes (the number of gene copies). A large number of transcriptome studies reported that in the early stages after genome doubling *per se*, synthesized autopolyploids expressed only a small fraction of protein coding genes (PCGs) and long non-coding RNA (lncRNA) of all loci at different level relative to the diploid, named genome-dosage effect (also referred to dosage response) (Martelotto et al., 2005; Stupar et al., 2007; Parisod et al., 2010; Yu et al., 2010; Allario et al., 2011; Del Pozo and Ramirez-Parra, 2014; Xiao et al., 2019). However, the putative mechanisms for this process is largely unknown. Autopolyploids, especially artificial lines, ruling out disturbances from incompatible genomes, offer an extraordinary opportunity to understand mechanisms of genome-dosage effect.

Doubling a set of chromosome cause “genome shock,” associated with dramatic changes in the epigenetic modifications (Chen, 2013; Song and Chen, 2015). DNA methylation provides an effective mean for a polyploid cell to overcome “genomic shock” caused by WGD (Chen, 2013). Cytosine methylation is a common feature of epigenetic regulation that influences many molecular processes, including embryogenesis (Rival et al., 2013), transposable elements (TEs) activity (Saze and Kakutani, 2011; Ibarra et al., 2012), and gene expression (Zilberman et al., 2007). Plant genomes are often methylated in CG, CHG, and CHH (H = A, T, or C) contexts (Law and Jacobsen, 2010). Many studies in allopolyploid crop species indicated that gene expression is altered more by interspecific hybridization than by polyploidization (Shaked et al., 2001; Madlung et al., 2002; Kraitshtein et al., 2010). However, to date, there are almost no reports on DNA methylation variations to reveal the impact on PCGs expression responding to autotetraploidization except in rice (*Oryza sativa* ssp. Indica) (Zhang et al., 2015).

The complement of TEs within any one genome typically includes both class I retrotransposon and class II DNA transposons (Feschotte et al., 2002). TEs make up a substantial fraction of mature lncRNA transcripts, they are also enriched in the vicinity of lncRNAs, where they frequently contribute to their transcriptional regulation (Kapusta et al., 2013; Wang et al., 2016). Zhao et al. (2018) reported that demethylated *LINEs*/TEs might distinctively impact lncRNAs expression in polyploid cotton interspecific F_1_ hybrid in genomic shock caused by interspecific hybrid and WGD. Nevertheless, the impact on the lncRNAs expression of whole genome caused by WGD, especially TE-overlapped lncRNAs, remains largely unknown.

Cassava (*Manihot esculenta* Crantz), a perennial shrub of the Euphorbiaceae family, is one of the most important food and energy crops in the world and is ranked the third most consumed carbohydrate source and for millions of people in the tropics (Raven et al., 2006). The cassava genome is highly heterozygous because of its outcrossing nature and broad tropical distribution (Fregene et al., 2003; Siqueira et al., 2010). Previously, we obtained an autotetraploid cassava (4x) from the diploid cultivar (2x), which was produced by colchicine-induced (Zhou et al., 2017). Here, we generated integrated maps of methylomes and lncRNAomes in autotetraploid cassava and its donor parent, both of which were independently clonally propagated for 2 years, in order to evaluate the short-term impact of intraspecies genome duplication on the expression of PCGs and lncRNAs of whole genomes.

## Materials and Methods

### Plant Materials

An autotetraploid cassava line (2n = 4x = 72) was artificially created by the cultivar “Xinxuan 048” (2n = 2x = 36) using aqueous colchicine solution (Zhou et al., 2017). The ploidy levels of the generated autoploid plants were detected by the flow cytometry analysis, and then chromosome counting in root-tip cells confirmed the results of flow cytometry analysis by Zhou et al. (2017). The plant architecture screening was carried out for the first two generations. Stem-propagated plants from each 2x and 4x cassava were sown and grown in plastic pots with a photoperiod of 16/8 h (day/night) in the greenhouse of Guangxi Academy of Agricultural Sciences (GXAAS). At ∼60 days after planting, 2x and 4x cassava plants have equal numbers of leaves at this stage, the fifth leave (counting from the top of the plant) of nine individual plants of each cytotype were sampled. Three plants each biological replicate were included and each cytotype has triplicates. The collected leaves were immediately frozen in liquid nitrogen, and stored at −80°C until total DNA and RNA extractions were performed.

### DNA Extraction

Genomic DNA was extracted according to a plant genomic DNA kit [TIANGEN BIOTECH (Beijing) Co., Ltd., China, code number: DP305] following the manufacturer’s instructions. After genomic DNAs were extracted from the samples, DNA concentration and integrity were detected by NanoPhotometer^®^ spectrophotometer (IMPLEN, CA, United States) and Agarose Gel Electrophoresis, respectively.

### Library Construction, Sequencing, and Data Filtering

The DNA libraries for Bisulfite sequencing (BS-seq) were prepared. Briefly, genomic DNAs were fragmented into 100–300 bp by Sonication (Covaris, MA, United States) and purified with MiniElute PCR Purification Kit (QIAGEN, MD, United States). The fragmented DNAs were end repaired and a single “A” nucleotide was added to the 3′ end of the blunt fragments. Then the genomic fragments were ligated to methylated sequencing adapters. Fragments with adapters were bisulfite converted using Methylation-Gold kit (ZYMO, CA, United States), unmethylated cytosine is converted to uracil during sodium bisulfite treatment. Finally, the converted DNA fragments were PCR amplified and sequenced using Illumina HiSeq™ 2500 by Gene Denovo Biotechnology Co. (Guangzhou, China).

To get high quality clean reads, the reads containing more than 10% of unknown nucleotides and low quality reads containing more than 40% of low quality (*Q*-value ≤ 20) bases were removed from the raw reads generated from Illumina HiSeq™ 2500.

### Transposable Element Annotation

Transposable elements were annotated by running RepeatMasker^1^ against a cassava reference genome sequence (v6.1^2^). In detail, Tandem repeats finder (Benson, 1999) software was used to predict tandem repeats. Prediction method of Interpersed repeat was as following: (1) Considering some repeat sequences often have specific sequence characteristics, such as long terminal repeats (LTRs) transposon, which is characterized by symmetric LTR at both ends, we predicted LTR transposons through LTR_FINDER (Xu and Wang, 2007), Helitron transposon by Helitroscanner (Xiong et al., 2014), MITE transposon by MITE-Hunter (Han and Wessler, 2010). LINE by MGEscan-non-LTR (Lee et al., 2016). (2) Since the repeat sequence has multiple copies in the genome, multiple copies of the repeat sequence in the genome can be found through mutual alignment within the genome sequence. First, three softwares PILER (Edgar and Myers, 2005), RepeatScout (Price et al., 2005), and RepeatModeler (Bao and Eddy, 2002) were used to obtain preliminary *de novo* prediction results, and then sequences classified as DNA and LINE are extracted and merged into one file. The redundancy of the filtering sequence itself, and the filtering standard identity >90%. (3) Homology construction based on the principles of structure prediction and *de novo* (*de novo*), a repeat sequence database was constructed, which was combined with Repbase database as the final repeat sequence database. Then RepeatMasker (Tarailo-Graovac and Chen, 2009) software was used to predict the repeat sequence of sequencing data based on the constructed repeat sequence database. Collectively, a dataset of 12,592 TEs was used for further analysis (Supplementary Table 1).

### Methylation Level Analysis

The obtained clean reads were mapped to cassava reference genome using BSMAP software (Xi and Li, 2009) (version: 2.90) using default setting. We used a custom Perl script to call methylated cytosines (mC) and these methylated cytosines were tested with the correction algorithm described in Lister et al. (2008). The overall methylation levels were calculated using a BSMAP package script according to the ratio of reads (mC)/[reads (mC) + reads (non-mC)]. The methylation level was calculated based on methylated cytosine percentage in the whole genome, in each chromosome and in different regions of the genome for each sequence context (CG, CHG, and CHH). To assess different methylation patterns in different genomic regions, the methylation profile at flanking 2-kb regions and PCGs (lncRNAs or TEs) body was plotted based on the average methylation levels for each window.

### Analysis of Differentially Methylated Regions

To determine the differentially methylated regions (DMRs) between 2x and 4x cassava cytotypes, the minimum read coverage to call a methylation status for a base was set to 4. DMRs for CG, CHG, and CHH context according to different criteria: (1) for all C, numbers in a window ≥ 20, absolute value of the difference in methylation ratio ≥ 0.2, and *q* ≤ 0.05; (2) for CG, numbers of GC in a window ≥ 5, absolute value of the difference in methylation ratio ≥ 0.25, and *q* ≤ 0.05; (3) for CHG, numbers in each window ≥ 5, absolute value of the difference in methylation ratio ≥ 0.25, and *q* ≤ 0.05; (4) for CHH, numbers in a window ≥ 15, absolute value of the difference in methylation ratio ≥ 0.25, and *q* ≤ 0.05.

### LncRNA-seq and Data Analysis

Transcriptome sequencing was performed with the same leaf used for BS-seq for each of the diploid and autopolyploid cassava. Total RNA was extracted from 100 mg of tissue using TRIzol reagent (Invitrogen) according to the manufacturer’s instructions. The rRNAs were removed with Ribo-Zero rRNA Removal Kit (Plant) (Illumina, item number: MRZPL1224) to retain mRNAs and ncRNAs. The enriched mRNAs and ncRNAs were fragmented into short fragments by using fragmentation buffer and reverse transcribed into cDNA with random primers. Second-strand cDNA were synthesized by DNA polymerase I, RNase H, dNTP (dUTP instead of dTTP) and buffer. Next, the cDNA fragments were purified with QIAquick PCR extraction kit (QIAGEN, Venlo, Netherlands), end repaired, poly(A) added, and ligated to Illumina sequencing adapters. Then UNG (Uracil-N-Glycosylase) was used to digest the second-strand cDNA. The digested products were size selected by agarose gel electrophoresis, PCR amplified, and sequenced using Illumina HiSeq™ 4000 (PE150) by Gene Denovo Biotechnology Co. (Guangzhou, China). After removing sequences containing adapters, poly-N and low quality reads, clean reads were aligned to the cassava reference genome (v6.1, see text footnote 2) by HISAT2 (version 2.1.0) with “-rna-strandness RF” and other parameters set as a default (Kim et al., 2013). The reconstruction of transcripts was carried out using Stringtie (version 1.3.4), which together with HISAT2 (Pertea et al., 2015, 2016). To identify novel genes, all of the reconstructed transcripts were mapped to the cassava reference genome and were divided into 12 categories by using Cuffcompare (Trapnell et al., 2012). Gene abundances were quantified using RSEM (v 1.2.19) (Li and Dewey, 2011) and PCG- and lncRNA-expression levels were normalized using FPKM (Fragments Per Kilobase of transcript per Million reads). Two softwares CPC (version 0.9-r2) and CNCI (version 2) (Kong et al., 2007; Sun et al., 2013) were used to assess the protein-coding potential of novel transcripts by default parameters. The intersection of both non-protein-coding potential results and non-protein annotation results were chosen as lncRNAs.

Differential expression analysis of PCGs and lncRNAs was performed by DESeq2 (Love et al., 2014) software between two different cytotypes. We used a false discovery rate (FDR) < 0.05 and fold change ≥ 2 as the thresholds to determine significant differences in PCG and lncRNA expression.

## Results

### Single Base-Resolution Maps of DNA Methylation for Diploid and Autotetraploid Cassava

In order to investigate the potential role of DNA methylation in response to autotetraploidy, the methylomes of diploid (2x) and autotetraploid (4x) cassava leaves were decoded and analyzed. Genome mapping analysis showed that overall, 113,078,644 (73.50%), 109,732,951 (73.44%), and 111,434,206 (74.25%) clean reads from the three diploid biological replicates, and 119,723,975 (74.70%), 96,864,854 (75.06%), and 110,185,024 (75.65%) clean reads from the three autotetraploid biological replicates, were mapped to the genome. The sequence depth of all the samples were more than 24×. More than 99% of cytosines were altered, which indicated that a high rate of conversion (Supplementary Table 2). In addition, Pearson correlation coefficients between the three replicates of 2x and 4x were found to be between 0.96 and 0.97 (Supplementary Figure 1). All the data indicated that the quality of sequencing was satisfactory to subsequent analysis. 2x and 4x cassava showed no significant differences in overall methylation levels, regardless of sequence context, across all the sequenced C sites, the 2x and 4x genome presented 67.09 and 66.94% mCGs, 49.03 and 48.67% mCHG, and 5.88 and 5.74% mCHH, respectively (*T*-test, *P* > 0.05) (Supplementary Figure 2). At the chromosome-scale scale, it was discovered that the methylation levels of three contexts were all predominantly highly pericentromeric heterochromatin regions and methylation levels of all three contexts in 2x and 4x cassava were similar to one another (Supplementary Figure 3).

### Landscape of Protein Coding Genes, Long Non-coding RNAs, and Transposable Elements Methylation

**FIGURE 1:**
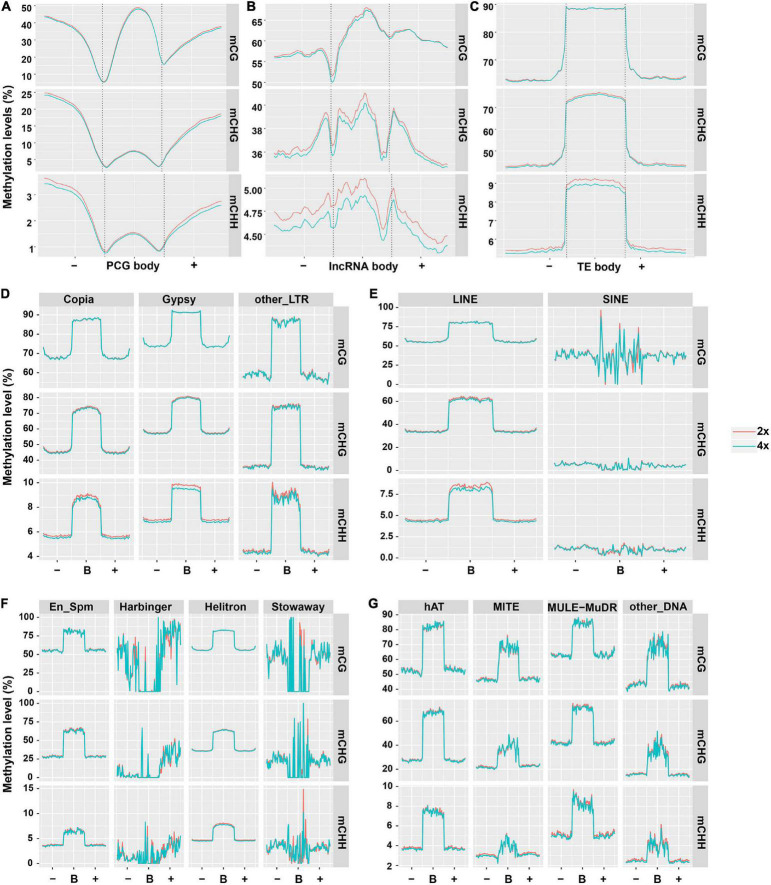
DNA methylation patterns in PCGs, lncRNAs, and TEs. Average methylation level distributions over **(A)** PCGs, **(B)** lncRNAs, and **(C)** TEs. Average methylation level distribution over **(D,E)** class I TEs and **(F,G)** class II TEs. The average level for each 100-bp interval is plotted. The dashed lines for the PCG, lncRNA, and TE regions indicate the transcriptional start (left) and end (right) sites.

To characterize the DNA methylation patterns in different cassava genomic regions, we constructed the methylation profiles within PCGs, lncRNAs, and TEs, together with 2-kb regions flanking the genes, using the same lengths cut-off. Methylation patterns in PCGs in our study are generally similar to those in soybean (Song et al., 2013), the peak of CG methylation in the PCGs body was higher than that in the flanking regions (Figure 1A). In contrast, CHG methylation had a similar tendency to CHH methylation in that obviously lowered levels were observed in body regions compared with upstream or downstream regions (Figure 1A). There were no methylation differences in CG and CHG contexts between PCGs from 2x and 4x cassava (*T*-test, mCG *P*-value = 0.390, mCHG *P*-value = 0.451). The difference methylation level of mCHH (*T*-test, *P*-value < 0.05) could be negligible because the highest methylation level of mCHH is only about 1.5% in PCG bodies in both 2x and 4x cassava. The results suggested that WGD may not have widespread influence over methylation state of PCG bodies. The methylation state of lncRNA in all three contexts were higher than those of PCG, moreover, 4x cassava had decreased CG, CHG, and CHH methylation levels relative to 2x across lncRNA body regions and flanking regions (*T*-test, mCG *P*-value = 0.151, mCHG *P*-value = 0.018, mCHH *P*-value = 0.044) (Figure 1B), suggesting WGD may have widespread influence on methylation levels of lncRNAs bodies together with 2-kb flanking regions in CHG and CHH contexts, rather than CG context. As is seen in Figure 1C, consistent with rice and other plant species (Lister et al., 2009; Feng et al., 2010; Zemach et al., 2010), average methylation level of TEs was much higher than that of PCGs and lncRNAs. 4x cassava had decreased methylation levels in CHG and CHH contexts relative to 2x cassava in TE bodies, there is no significance difference of mCG methylation level in TE bodies between 2x and 4x cassava (*T*-test, mCG *P*-value = 0.455, mCHG *P*-value = 1.59E−13, mCHH *P*-value = 1.58E−93) (Figure 1C).

Consequently, we detected the methylation differences of the two classes of TEs. All the 13 orders of TEs had unique average methylation distribution, and exhibited hypermethylation state in body regions than flanking regions in all three contexts. SINE, Stowaway, Harbinger and other_DNA TE types make up a small fraction of the genome (<0.2% each) and were not considered for further analysis (Supplementary Table 3). 4x cassava exhibited hypomethylation levels in mCHG and mCHH sites in body regions of Copia and Gypsy, respectively (*T*-test, Copia mCHG *P*-value = 0.042, mCHH *P*-value = 1.41E−06; Gypsy mCHG *P*-value = 3.86E−05, mCHH *P*-value = 2.97E−40) (Figures 1D,E). We also found that body regions of Helitron and hAT from 4x cassava had increased CG methylation levels (*T*-test, Helitron *P*-value = 0.027, hAT *P*-value = 0.032). Moreover, body regions of *MITE* from 4x cassava were hypermethylated in CG and CHH contexts (mCG *P*-value = 0.041, mCHH *P*-value = 0.030) (Figures 1F,G).

Further, we found that TEs accounted for 60% of the cassava genome, class II TEs were inclined to localize in euchromatin regions near PCGs (Wang et al., 2014; Bredeson et al., 2016). On the other hand, TEs tended to locate in the intronic sequences or the flanking regions of the PCGs, however, only a small portion of PCGs have TEs in their introns (Wang et al., 2014, 2015). In this case, we speculated that genome-wide changes of TEs methylation levels may affect expression or activities of neighboring PCGs that were inserted and surrounded by class II TEs after WGD, given that WGD may not have widespread influence over methylation state of PCG bodies (Figure 1A). On the other hand, TE-overlapped lncRNAs made up 60% of all detected lncRNAs (Supplementary Figure 4A), and class I TEs-overlapped lncRNA accounted for 54% (Supplementary Figure 4B), supporting the statement that substantial portion of lncRNAs are either derived from TEs or contain TEs remnants (Kapusta et al., 2013; Wang et al., 2016, 2017). Consequently, we hypothesized that genome-wide alteration of class I TEs methylation levels may affect nearby lncRNAs expression as a result of autotetraploidization. Therefore, it was sensible and necessary to combine TE methylation and PCG and lncRNA expression to examine the epigenetic responses to WGD.

### Gene Methylation Is Associated With Gene Activity

**FIGURE 2:**
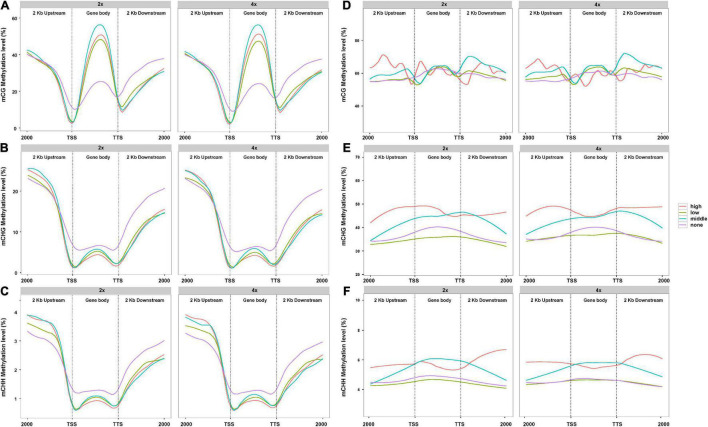
Association between DNA methylation level and PCGs expression in 2x and 4x cassava. Effect of DNA methylation of **(A)** mCG, **(B)** mCHG, and **(C)** mCHH on genome-wide PCGs expression. Effect of DNA methylation of **(D)** mCG, **(E)** mCHG, and **(F)** mCHH on global lncRNAs expression.

In view of the difference of methylation between 2x and 4x cassava after genome doubling, we attempted to understand whether PCG- and lncRNA-expression levels were influenced by DNA methylation. A total of 33,030 PCGs and 13,517 lncRNAs analyzed from the lncRNA-seq data in “Xinxuan 048” were divided into four quartiles from high-expressed group, low-expressed group, middle-expressed group, and none-expressed group based on their expression levels according to the criteria of Yan et al. (2018). As is shown in Figure 2A, in PCGs body regions, the highest mCG methylation levels were not detected in the highly expressed PCGs, but instead in those that are middle highly expressed in 2x and 4x cassava. In PCGs body, PCGs with no expressed displayed the lowest mCG methylation levels. High-expressed PCGs displayed the lowest CHG methylation levels, middle-expressed PCGs showed the second highest CHG methylation levels, and PCGs with non-expressed displayed the highest methylation level in the 2x and 4x cassava (Figure 2B). The same correlation was found between mCHH methylation and PCGs activity (Figure 2C). The results suggested that mCG levels in the PCGs body regions were positively correlated to PCGs expression level, whereas there is a negative correlation between CHG and CHH methylation levels and PCG expression in 2x and 4x cassava. As is shown in Figure 2D, there was no clear relationship between DNA methylation of mCG and lncRNA expression. In lncRNAs body together with upstream regions and downstream regions, lncRNAs with high-expressed showed the highest CHG methylation levels, middle-expressed lncRNAs displayed the second highest CHG methylation levels, and lncRNAs with non-expressed displayed almost the same relative low methylation level as that of the low-expressed lncRNAs in 2x and 4x, respectively (Figure 2E). The highest mCHH methylation levels were not detected in the highly expressed lncRNAs, but instead in those that are middle highly expressed in 2x and 4x cassava (Figure 2F). Together, though no correlation was observed between CG methylation and lncRNA-expression levels, CHG methylation levels in lncRNA body were positively correlated with lncRNA expression.

### Transposons With Changed DNA Methylation Caused by Whole Genome Duplication Altered the Expression of Nearby Protein Coding Genes and Long Non-coding RNA

To better understand gene-expression pattern influenced by DNA methylation and the relationships with TEs, total RNA was extracted from leaves to generate lncRNA-seq data. Comparison of gene-expression level between 2x and 4x cassava indicated that only 359 PCGs and 402 lncRNAs were differentially expressed, respectively. That is, relative to the diploid, few PCGs and lncRNAs were significantly differentially expressed in 4x cassava despite doubled gene dosage (Wilcoxon rank sum test, PCG *P*-value = 0.5062, *n* = 33,030; lncRNA *P*-value = 0.003689, *n* = 13,517) (Supplementary Figure 5). Compared with 2x cassava, there were 173 PCGs up-regulated, and there were 186 PCGs down-regulated (Supplementary Figure 5A); there were 204 lncRNAs up-regulated, and 198 lncRNAs down-regulated in 4x cassava (Supplementary Figure 5B).

**FIGURE 3:**
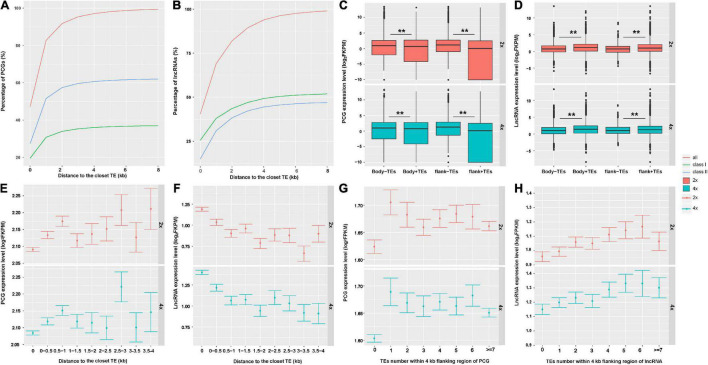
Transposable elements altered expression of neighboring PCGs and lncRNAs in cassava. The percentage of **(A)** PCGs and **(B)** lncRNAs inserted by TEs in their bodies and within 8-kb flank regions. The expression levels of **(C)** PCGs and **(D)** lncRNAs inserted by TEs or not. “+” means TEs inserted in this region; *P-value < 0.05; **P-value < 0.01. **(E)** PCGs and **(F)** lncRNAs expression level related to the distance to the closest TE. “0” indicates genes overlapped with TEs in body regions. Error bars indicates SEM. The expression levels of **(G)** PCGs and **(H)** lncRNAs related to the number of neighboring TEs.

Consequently, we further asked whether and how TEs may affect expression of neighboring genes that were involved in WGD-induced variation in cassava. As is seen in Figure 3A, approximately 48% of PCGs had TEs inserted into their bodies and most of the TE insertions were class II. About 28 and 62% of PCGs were inserted by DNA transposons within bodies and 8-kb flanking regions, respectively. About 40% of lncRNAs had TEs into their bodies, 80% of lncRNAs were inserted by TEs within 2 kb, 94% lncRNAs overlapped with TEs within 4-kb flanking regions. Most of the TEs inserted into lncRNAs were retrotransposons, 25 and 52% of lncRNAs had retrotransposons insertion into their bodies and within 8-kb flanking regions, respectively (Figure 3B). Moreover, we found that in 2x and 4x cassava, PCGs without neighboring TEs were expressed at higher levels than those inserted or surrounded by TEs [*T*-test, Body-TE *P*(2x) value = 2.2E−16, *P*(4x) value = 2.2E−16; Flank-TE *P*(2x) value = 2.2E−16, *P*(4x) value = 2.2E−16] (Figure 3C). The expression levels of lncRNAs inserted or surrounded by TEs were higher than those without nearby TEs in 2x and 4x cassava [*T*-test, Body-TE *P*(2x) = 2.2E−16, *P*(4x) = 1.489E−7; Flank-TE *P*(2x) = 2.2E−16, *P*(4x) = 0.0057] (Figure 3D). The average PCG-expression level was positively correlated with the distance to the closest TE (Figure 3E), for both 2x and 4x cassava, in contrast, the average lncRNA-expression level was negatively correlated with the distance to the closet TE (Figure 3F). In addition, the average PCG-expression level was negatively correlated with the number of TEs within 4-kb flanking regions (Figure 3G), however, lncRNA-expression level was positively correlated with the number of TEs within 4-kb flanking regions in 2x and 4x cassava (Figure 3H). Collectively, these results indicated that PCG-expression levels were suppressed by the abundance and physical distances from adjacent TEs, and lncRNA-expression levels were activated by the abundance and physical distances from proximal TEs in cassava.

**FIGURE 4:**
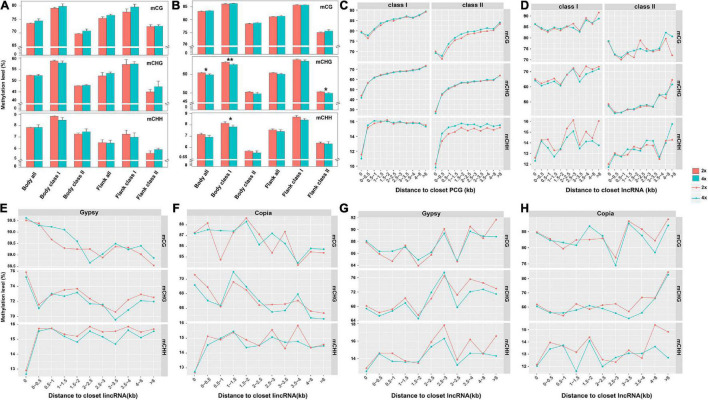
DNA methylation level related to the distance from the closest PCG and lncRNA. DNA methylation of TEs from **(A)** PCG and **(B)** lncRNA body of whole genome, together with flanking 4-kb regions. Upper represented mCG, middle represented mCHG and lower presented mCHH. TE methylation level related to the distance from the closest **(C)** PCG and **(D)** lncRNA. **(E)** *Gypsy* and **(F)** *Copia* methylation level related to the distance from the closest lincRNA. **(G)** *Gypsy* and **(H)** *Copia* methylation level related to the distance from the closest lncRNA. *P-value < 0.05; **P-value < 0.01.

Considering expression level of adjacent genes were negatively correlated with the state of methylated TEs in *Arabidopsis* and rice (Hollister and Gaut, 2009; Zhang et al., 2015), we compared DNA methylation of TEs from the whole genome, gene body, and flanking 4-kb regions between 2x and 4x cassava. Methylation of class II TEs, for both PCGs and lncRNAs, were lower than that of class I TEs for all three contexts, and methylation levels of TEs or class II TEs for PCGs in 4x cassava were higher than those of 2x except for flanking TEs in CHH context (*T*-test, Supplementary Table 4 and Figure 4A). Compared with 2x cassava, TEs inserted in lncRNA bodies from 4x cassava exhibited hypomethylation level in the CHG context (*T*-test, Supplementary Table 5). Class I TEs inserted in lncRNA bodies or surrounding lncRNAs from 4x cassava showed hypomethylation state exhibited hypomethylation level in the CHG and CHH context (*T*-test, Supplementary Table 5 and Figure 4B).

We divided PCG-flanking regions into different bins and compared methylation levels between two TE classes located within them (Figure 4C). Obviously, methylation levels of class II TEs nearby PCGs were lower than that of class I TEs in all three contexts. The CG methylation levels of class II TEs gradually decreased with increased distances from PCGs, and the valley of CG methylation levels of TEs appeared within 0.5-kb flanking regions. For mCHG and mCHH sites, class II TEs methylation levels gradually increased with increased distances from PCGs. Notably, for mCHG and mCHH sites, 4x cassava exhibited decreased class II TEs methylation levels in PCGs body regions, consisting with hypomethylation levels of PCGs body regions in CHG and CHH contexts in 4x cassava (Figure 1A). In contrast, 4x cassava exhibited hypermethylation in CG and CHH contexts in PCG-flanking regions of class II TEs. The annotation of the hypermethylation CG and CHH level of class II TEs related to the distance from the closest PCGs which were not significant differentially expressed between 2x and 4x cassava were listed in Supplementary Tables 6, 7, respectively. To sum up, hypermethylation of class II TEs near PCGs in CG and CHH contexts, may be a direct response factor to overcome genomic shock following WGD in 4x cytotype cassava.

Parallelly, we divided lncRNA-flanking regions into different bins and compared methylation levels between two TE classes located within them (Figure 4D). Similar with that of PCGs, methylation levels of class II TEs nearby lncRNA in all three contexts were lower than those of class I TEs. The profile of methylation levels of class I TEs did not show always rising for lncRNAs, which was different from that of PCGs. The CHG methylation levels of class I TEs had the same dynamic change with that of CHH methylation levels with increased distances from lncRNAs excluding within 0- to 0.5-kb flanking regions. The CG methylation levels of class I TEs appeared to be higher in 4x cassava than that of 2x in 0- to 2.5-kb regions, however, CG methylation levels of class I TEs were lower in 4x in the flanking regions after 0- to 2.5-kb regions. Critically, in 4x cassava, class I TEs exhibited hypomethylation state in CHG and CHH contexts in lncRNA-flanking regions, with the exception that CHG methylation state in ∼1.5- to 3.0-kb lncRNA-flanking regions observed in 4x cassava was the same as that of 2x, and this result was consistent with the genome-wide methylation level of the five types of class I TEs (Figures 1D,E). The annotation of the hypomethylation CHG and CHH level of class I TEs related to the distance from the closest lncRNAs that were not significant differentially expressed between 2x and 4x cassava were listed in Supplementary Tables 8, 9, respectively. Taking together, reduction of CHG and CHH methylation levels of class I TEs nearby lncRNAs in 4x cassava may be a mechanism that suppressed expression of adjacent lncRNAs in 4x cassava with double alleles from the diploid line responding to genome-wide lncRNA dosage effects after WGD.

Further, we found that lincRNAs (or called intergenic lncRNA) accounted for the largest proportion (42%) of the whole lncRNAs (Supplementary Figure 6), the lncRNA loci contained more Gypsy and Copia segments than the other TEs at the exon and intron sequences together with 8-kb flanking regions in the cassava genome (Supplementary Figure 7). Gypsy showed the largest proportion of lncRNA-overlapped TEs, followed by Copia, due to its largest share of TEs in the cassava genome (Supplementary Figure 7). In order to understand whether the hypomethylated state of Gypsy or Copia was the effector overcoming genome shock in 4x cassava, we depicted the correlation diagram of the DNA methylation level of Gypsy and Copia related to the distance from the closest lncRNA and lincRNA in 2x and 4x cassava, respectively (Figures 4E–H). The results indicated that the profile of Gypsy methylation levels related to the closest lncRNA was found to be almost the same with that of class I TEs related to the closet lncRNA (Figure 4E).

### Comparisons of Differentially Methylated Regions Between 2x and 4x Cassava

**FIGURE 5:**
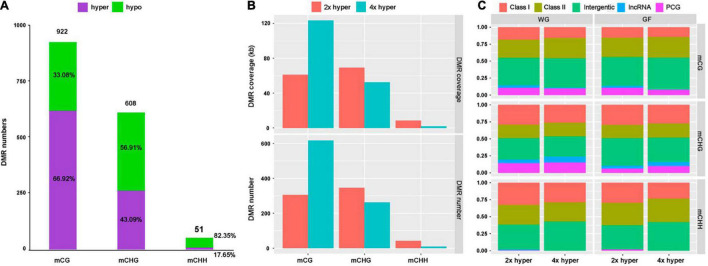
Differentially methylated region distribution. **(A)** DMR numbers of all three contexts and proportions of hyper- and hypo-methylated regions in all three contexts for 4x cassava compared with 2x. **(B)** Genome coverage and number of defined hypermethylation DMRs. **(C)** Distribution of hypermethylation DMRs in the whole-genome (WG) and gene-flanking 4-kb regions (GF).

To investigate the potential effect of WGD, we identified a total number of 922 CG, 608 CHG and 51 CHH DMRs (Figure 5A). A total of 64.92% of mCG sites were hypermethylated, while 43.09% of mCHG and 17.05% of mCHH sites exhibited hypermethylation in 4x cassava (Figure 5A).

At the whole-genome level, most DMRs came from the mCG context, hardly any CHH hyper-DMRs existed in 4x cassava (Figure 5B). The number of CG hyper-DMRs in 4x cassava was twice as much as in 2x cassava, while the number of CHG-DMRs and CHH-DMRs in 4x is less than that in 2x (Figure 5B). Genome-wide analysis showed that DMR of all three contexts were inclined to localize in intergenic and TEs regions rather than PCGs and lncRNAs (Figure 5C). Comparison with 2x cassava, 4x cassava exhibited CHG hyper-DMR and no CHH hyper-DMR within lncRNAs (Figure 5C). Analysis of PCG-flanking and lncRNA-flanking 4-kb regions revealed that the distribution of DMRs was similar to that throughout the whole genome (Figure 5C).

## Discussion

Many diploid crops such as rice (*O. sativa*), maize (*Zea mays*), soybean, poplar (*Populus trichocarpa*) are actually paleo-polyploids that have undergone ancient WGD events during their evolution (Jiang et al., 2013; Ren et al., 2018). Allopolyploid models have provided numerous clues to understand polyploidy formation, however, they can be confounded by the entanglement of both WGD and hybridization (Qin et al., 2021). Conversely, autopolyploids have made changes *via* WGD, ruling out disturbances from incompatible genomes. Newly resynthesized polyploids, which can be induced by colchicine, have enabled biologists to provide insights into genomic changes that occur in response to autotetraploidization.

Large-scale variations of DNA methylation have been found in allopolyploid plants (Lukens et al., 2006; Jiang et al., 2021; Yin et al., 2021). However, relatively little is known about the effects of autopolyploidization. Our findings showed that TEs methylation variations restrained expression of nearby PCGs and lncRNAs, indicating that it is an effective way to overcome genomic shock.

TEs, nearly ubiquitous in lncRNAs, represent a major force shaping the lncRNA repertoire of plants and animals, through their capacity to move and spread in genomes in a lineage-specific fashion (Loewer et al., 2010; Kapusta et al., 2013). In our study, most of the insertions into lncRNAs were retrotransposons in the cassava genome, which coincided with the results from previous studies (Golicz et al., 2018; Zhao et al., 2018; Lv et al., 2019). LncRNAs are highly hypomethylated in the CHG and CHH contexts, partially reflecting hypomethylation patterns in the CHG and CHH contexts of genome-wide class I TEs and class I TEs inserted or surrounding lncRNAs in 4x cassava. Therefore, the impact of polyploidization on DNA methylation pattern of class I TEs partially reflected the impact of WGD on methylation pattern of lncRNAs.

The previous results from *Arabidopsis* and rice, indicated that the number, distance of TEs, and methylation levels of TE affected nearby PCG activity (Hollister and Gaut, 2009; Wang et al., 2013; Zhang et al., 2015). Our study further confirmed that although there were 28% PCGs inserted by class II TEs within their bodies, most of PCGs were inserted by class II TEs within 4-kb flanking regions. 4x cassava displayed hypermethylation of class II TEs in CG context in PCG-flanking regions, which is consistent with increased genome-wide CG methylation level of class II TEs. It is probable that autopolyploidization acts as an effector that may stimulate TE activities. After short-term generations, neoautopolyploids survivors may gradually and adapt to WGD through many mechanisms, one of which could be the TEs hypermethylation or hypomethylation that would have effects on nearby PCGs or lncRNAs. In the case of lncRNAs, this would entail a fitness trade off between keeping TEs inactivated and suppressing proximal lncRNA-expression levels. These results suggested that genome-wide epigenetic silencing through DNA methylation of TEs might play prominent roles in adapting to genomic shock following WGD.

It should be noted, however, that though few genes are differentially expressed between diploid and tetraploid cassava, our data do not provide direct measures of gene dosage responses (Coate and Doyle, 2010, 2015; Loven et al., 2012). Thus, the extent to which the TE methylation changes we observe have restored diploid-like expression levels in the tetraploid are unknown. Additional transcriptomic analyses that enable estimation of expression per gene copy (Loven et al., 2012; Visger et al., 2019) will be required to address this open question. In our study, we have analyzed a single autotetraploid event, generated from a single cultivar, and this may not be representative of what would be observed in other, independent doubling events. For example, we found that increasing CG and CHH methylation levels of class II TEs near PCGs in 4x cassava may suppress expression of adjacent PCGs. In contrast, compared with the diploid rice, increasing methylation status of class II TEs in mCHG and mCHH sites restrained expression levels of nearby PCGs in an artificial synthesized rice tetraploid with a relatively stable genome (Zhang et al., 2015), and the methylation levels of class II TEs that surrounded PCGs in all three contexts were reduced in the colchicine induced autopolyploid switchgrass that was vegetatively propagated for 3 years (Yan et al., 2019). Therefore, we speculate that different species may adopt different methylation responses, which may be related with the stages after WGD. Additionally, CHH methylation of TEs may be more sensitive than CG and CHG methylation in modulating the expression of nearby genes. Overall, methylation level variation of TEs may be a beneficial strategy to help neoautopolyploids to conquer the early challenges caused by WGD in autopolyploid plants.

## Data Availability Statement

The original contributions presented in this study are publicly available. This data can be found here: https://www.ncbi.nlm.nih.gov/bioproject/, PRJNA728761.

## Author Contributions

LX and HY planned to conceive this project. LL and WZ provided valuable suggestions on the research design. LX and XS analyzed the data. SC prepared the samples. LX wrote the manuscript. HY revised the manuscript. All authors read and approved the final manuscript.

## Conflict of Interest

The authors declare that the research was conducted in the absence of any commercial or financial relationships that could be construed as a potential conflict of interest.

## Publisher’s Note

All claims expressed in this article are solely those of the authors and do not necessarily represent those of their affiliated organizations, or those of the publisher, the editors and the reviewers. Any product that may be evaluated in this article, or claim that may be made by its manufacturer, is not guaranteed or endorsed by the publisher.

## Footnotes

1
http://repeatmasker.org

2
https://phytozome.jgi.doe.gov/pz/portal.html#

## Supplementary Material

Supplementary Figure 1 Pearson correlation coefficients between and within groups.

Supplementary Figure 2 The methylation level of CG, CHG, and CHH sequence contexts in 2x and 4x cassava.

Supplementary Figure 3 Comparison of chromosome distribution between 2x and 4x cassava. Methylation level in 80-kb windows throughout chromosomes in the leaf tissue. The green and purple lines mean “+” strand, and the red and blue lines means “−” strand.

Supplementary Figure 4 The proportion of TE-overlapped lncRNA in this study.

Supplementary Figure 5 The percentage of differential expressed **(A)** PCGs and **(B)** lncRNAs between 2x and 4x cassava.

Supplementary Figure 6 The proportion of different types of lncRNAs detected in this study.

Supplementary Figure 7 Distribution of TEs in different lncRNAs features in cassava genome.
