# Supplementary figures and images for: DNA Methylome and LncRNAome Analysis Provide Insights Into Mechanisms of Genome-Dosage Effects in Autotetraploid Cassava
Source: Front Plant Sci. 2022 Jul 4;13:915056. doi: 10.3389/fpls.2022.915056 (PMC9289687; doi:10.3389/fpls.2022.915056)

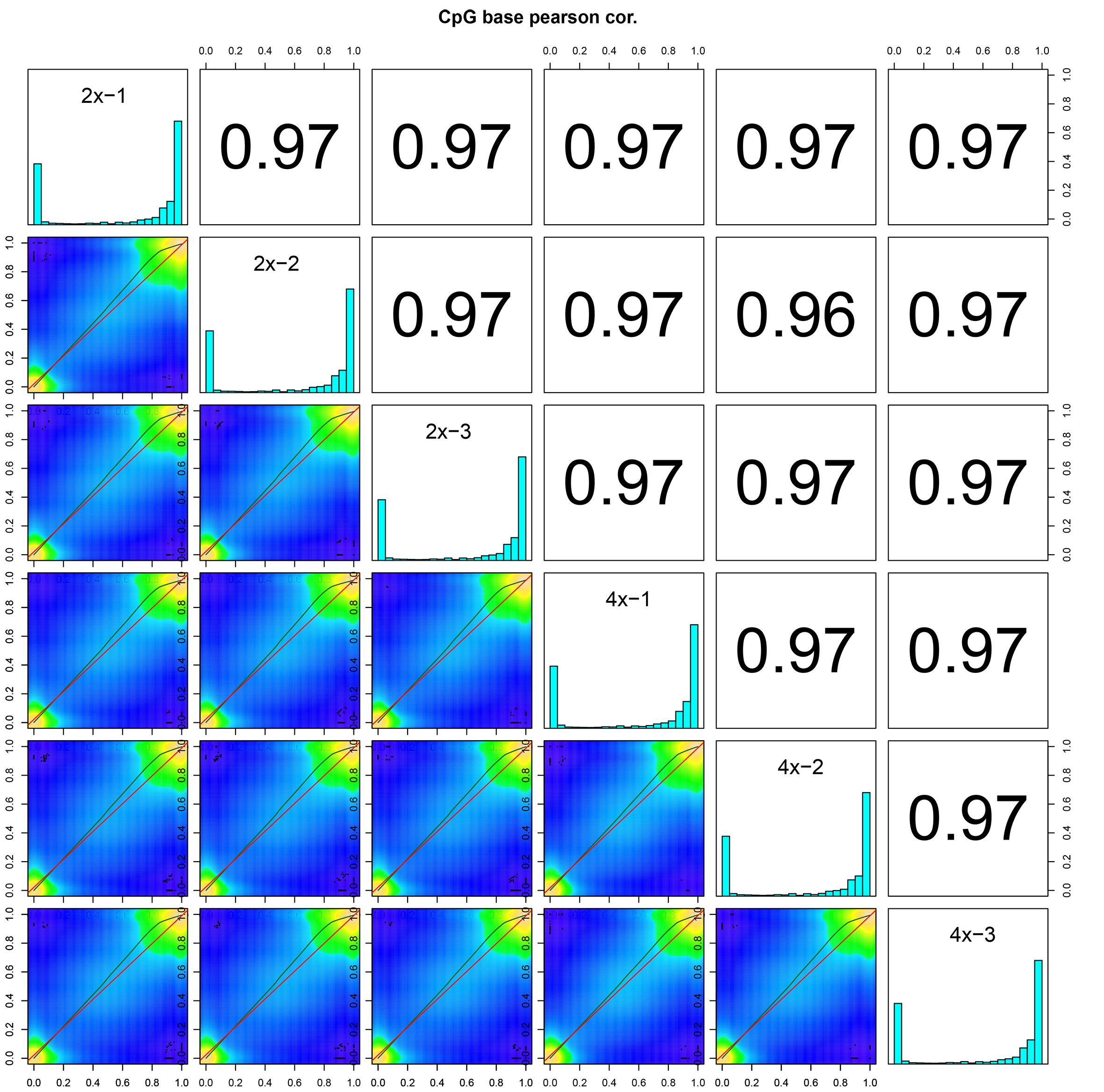

Supplement: Supplementary Figure 1 — Pearson correlation coefficients between and within groups. [file Image_1.TIF]

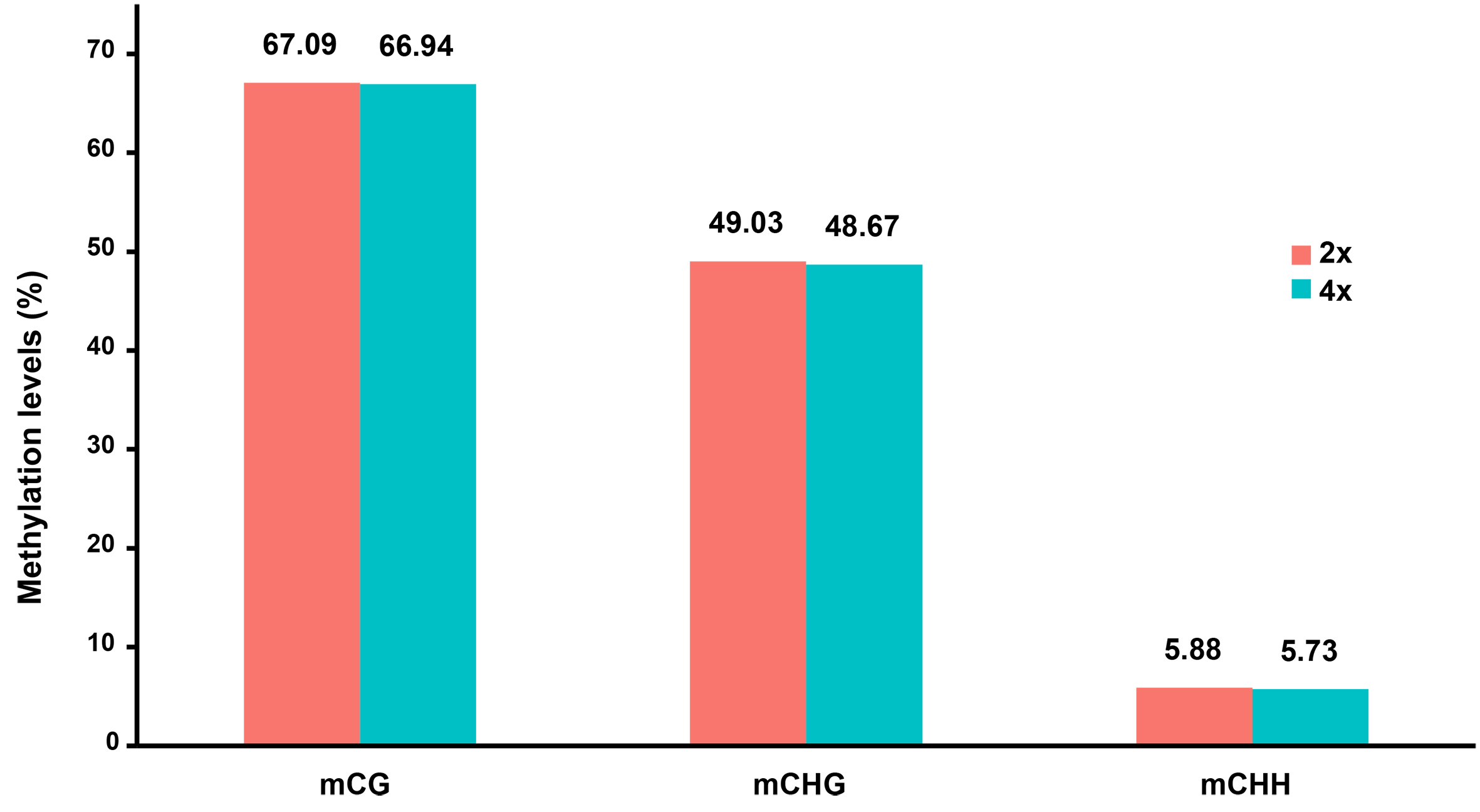

Supplement: Supplementary Figure 2 — The methylation level of CG, CHG, and CHH sequence contexts in 2x and 4x cassava. [file Image_2.TIF]

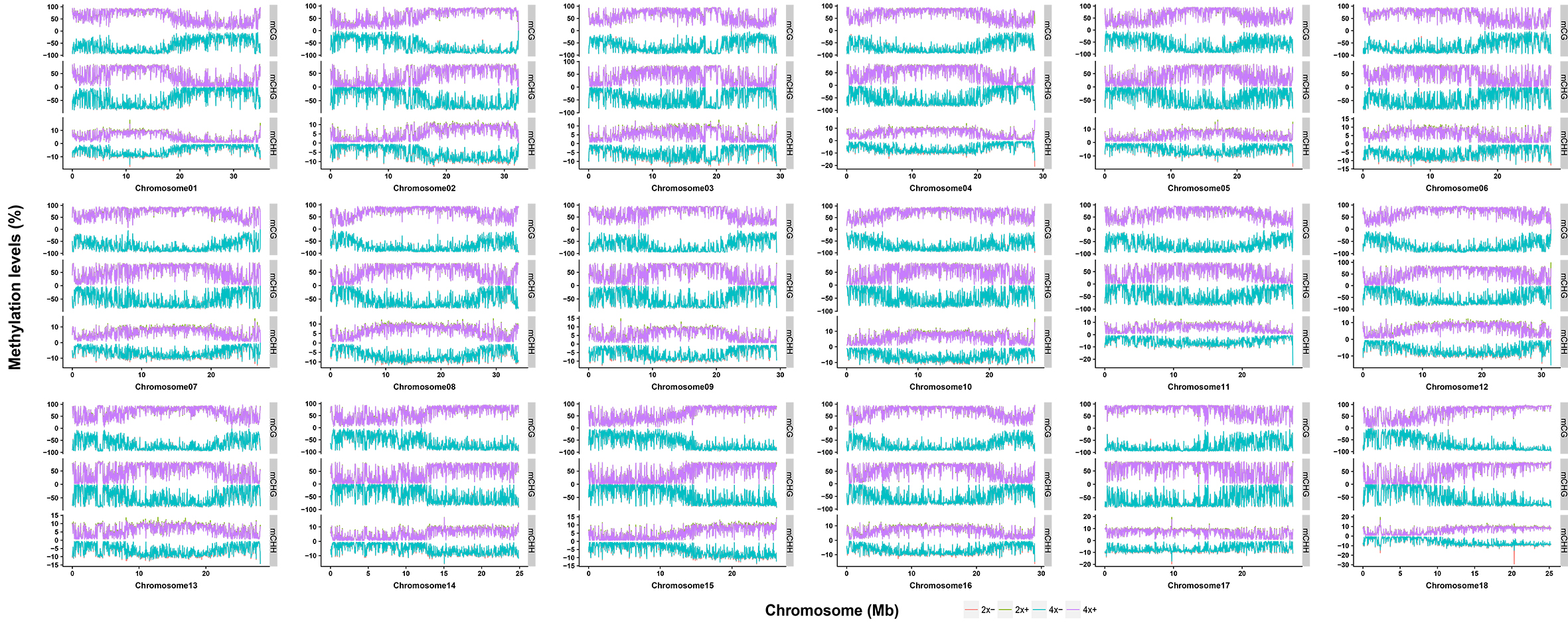

Supplement: Supplementary Figure 3 — Comparison of chromosome distribution between 2x and 4x cassava. Methylation level in 80-kb windows throughout chromosomes in the leaf tissue. The green and purple lines mean “+” strand, and the red and blue lines means “−” strand. [file Image_3.TIF]

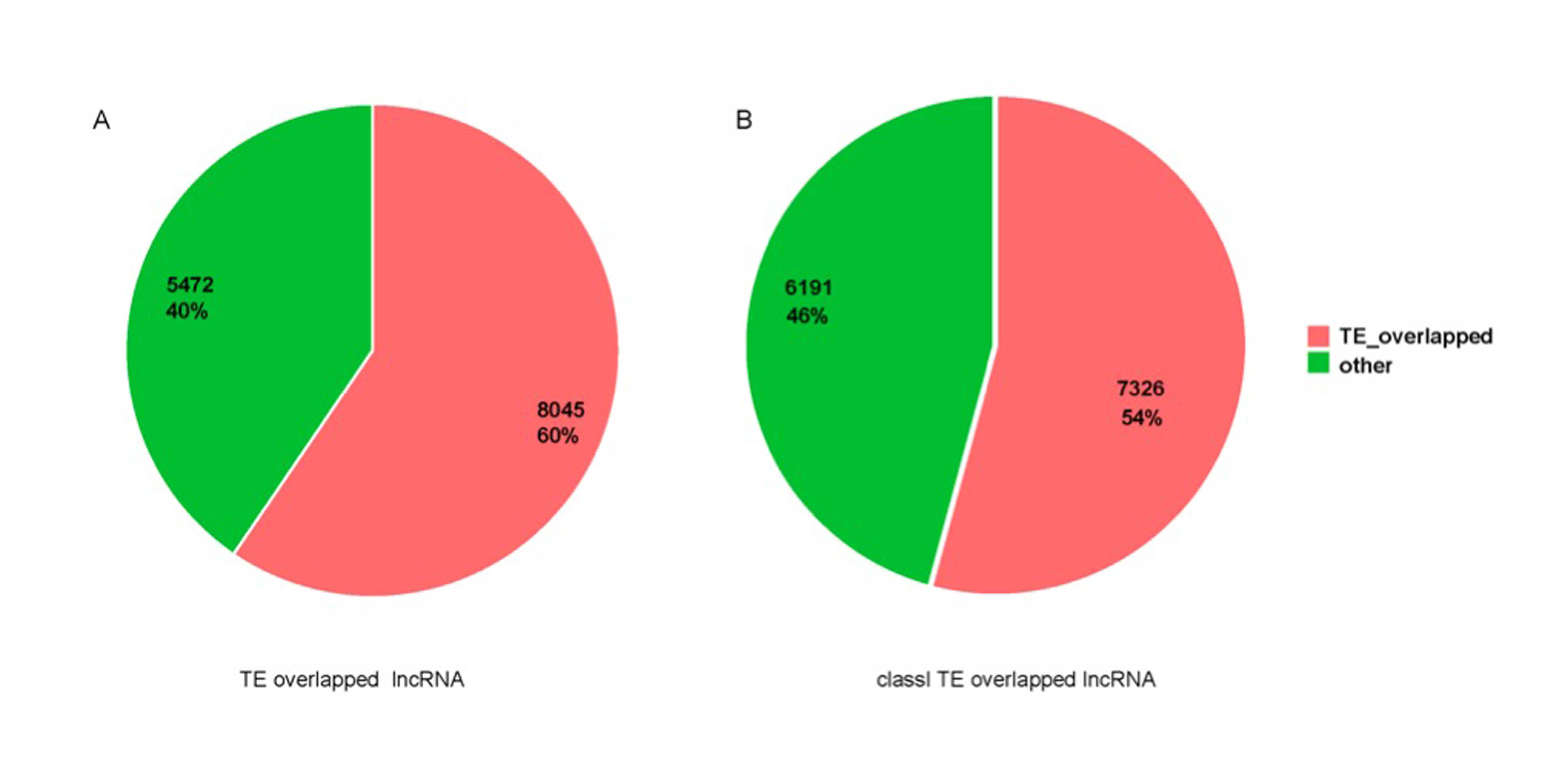

Supplement: Supplementary Figure 4 — The proportion of TE-overlapped lncRNA in this study. [file Image_4.TIF]

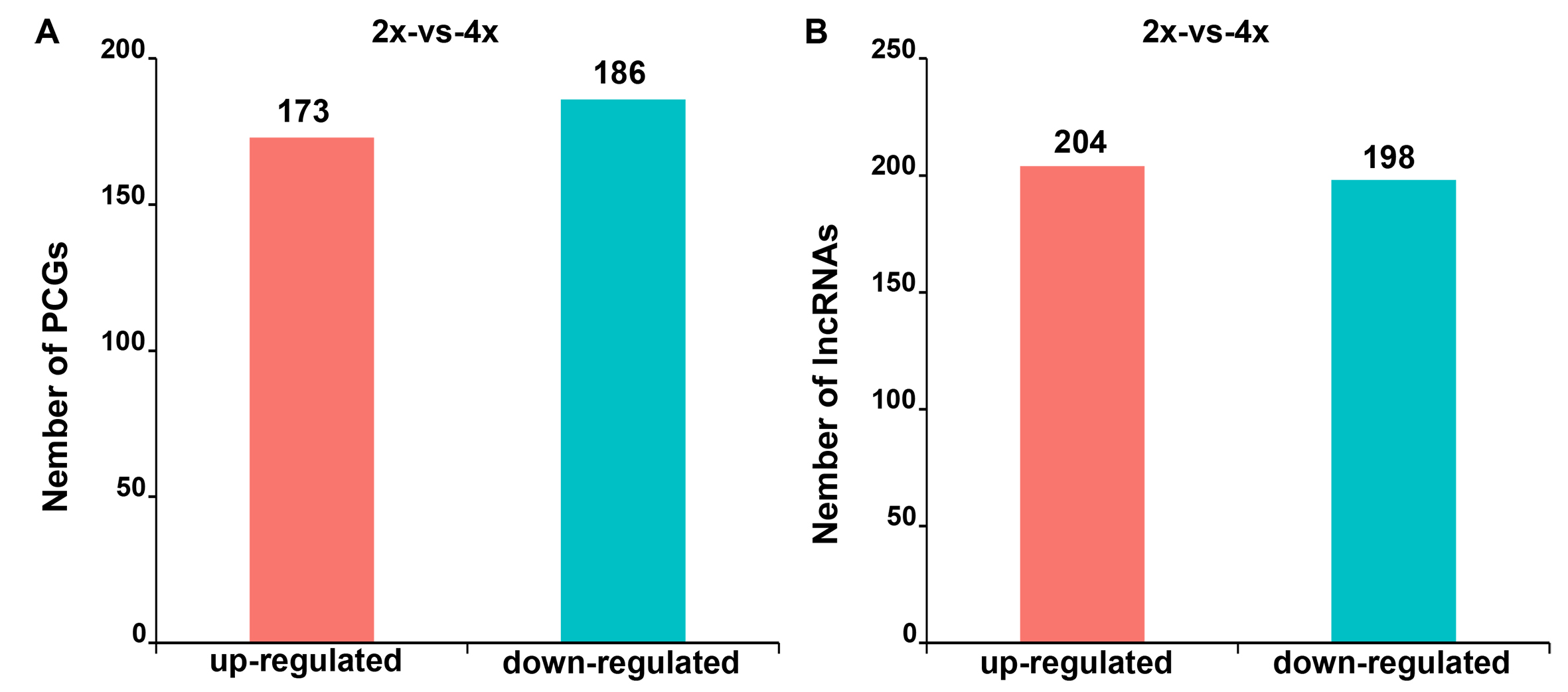

Supplement: Supplementary Figure 5 — The percentage of differential expressed (A) PCGs and (B) lncRNAs between 2x and 4x cassava. [file Image_5.TIF]

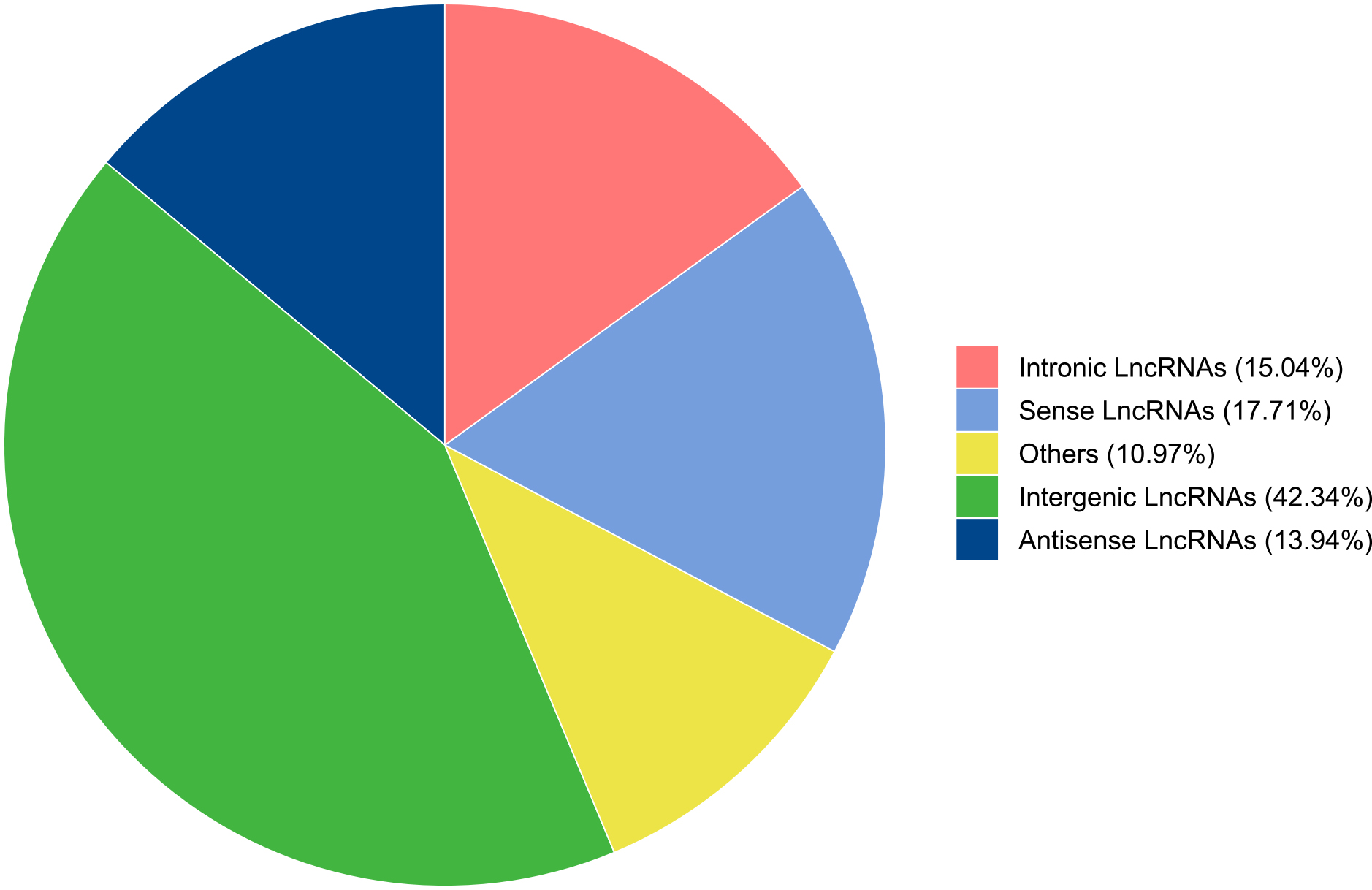

Supplement: Supplementary Figure 6 — The proportion of different types of lncRNAs detected in this study. [file Image_6.TIF]

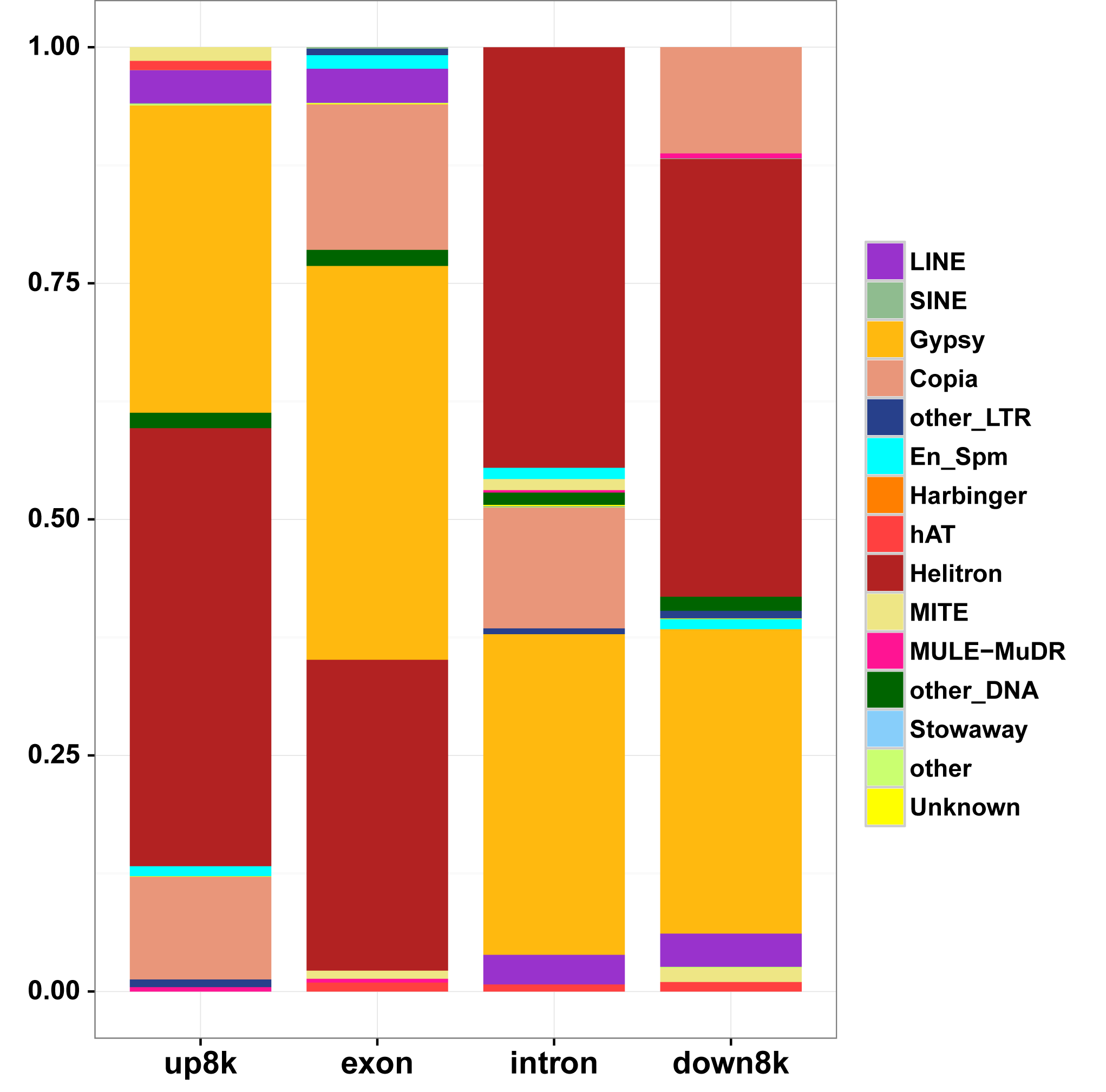

Supplement: Supplementary Figure 7 — Distribution of TEs in different lncRNAs features in cassava genome. [file Image_7.TIF]
