# Supplementary material for: DNA Methylome and LncRNAome Analysis Provide Insights Into Mechanisms of Genome-Dosage Effects in Autotetraploid Cassava
Source: Front Plant Sci. 2022 Jul 4;13:915056. doi: 10.3389/fpls.2022.915056 (PMC9289687; doi:10.3389/fpls.2022.915056)
Supplement: Supplementary file 8 [file Table_1.DOCX]

**Table S1. Summary of sequencing results of BS-seq in 2x and 4x cassava samples with three replicates.**

| **Samples** | **Total reads** | **Mapped reads** | **Mapped ratio** | **Sequence depth** | **BS conversion rate** |
| --- | --- | --- | --- | --- | --- |
| 2x-1 | 153855262 | 113078644 | 73.50 | 29.11 | 0.991638 |
| 2x-2 | 149413668 | 109732951 | 73.44 | 28.25 | 0.992340 |
| 2x-3 | 150074692 | 111434206 | 74.25 | 28.69 | 0.992500 |
| 4x-1 | 160278348 | 119723975 | 74.70 | 30.82 | 0.992358 |
| 4x-2 | 129051362 | 96864854 | 75.06 | 24.94 | 0.992445 |
| 4x-3 | 145660308 | 110185024 | 75.65 | 28.36 | 0.992297 |

BS conversion rate = 1 - methylation rate of Lambda DNA.
