# Supplementary material for: DNA Methylome and LncRNAome Analysis Provide Insights Into Mechanisms of Genome-Dosage Effects in Autotetraploid Cassava
Source: Front Plant Sci. 2022 Jul 4;13:915056. doi: 10.3389/fpls.2022.915056 (PMC9289687; doi:10.3389/fpls.2022.915056)
Supplement: Supplementary file 9 [file Table_2.DOCX]

**Table S2. The feature of the 13 TE subfamilies in the cassava genome.**

| **Type** | **TE_number** | **Length** | **Ratio^a^** |
| --- | --- | --- | --- |
| other_LTR | 4327 | 1656610 | 0.00284308 |
| MULE-MuDR | 2721 | 908475 | 0.00155913 |
| Gypsy | 225709 | 220582861 | 0.378565 |
| SINE | 271 | 24240 | 4.16E-05 |
| MITE | 7623 | 1612210 | 0.00276688 |
| Copia | 59195 | 35303334 | 0.0605877 |
| hAT | 4795 | 1617437 | 0.00277585 |
| Stowaway | 85 | 5817 | 9.98E-06 |
| other_DNA | 5306 | 573325 | 0.000983942 |
| LINE | 20251 | 8046191 | 0.0138089 |
| Harbinger | 14 | 856 | 1.47E-06 |
| En_Spm | 5218 | 1426093 | 0.00244746 |
| Helitron | 279655 | 100698761 | 0.17282 |

Note: ^a^ represented the percentage of the genome.
